# Dementia and Diabetes Mellitus: Association with Apolipoprotein E4 Polymorphism from a Hospital in Southern India

**DOI:** 10.1155/2012/702972

**Published:** 2012-06-03

**Authors:** Lakshmi Narayanan Kota, Bhagyalakshmi Mallapura Shankarappa, Prafulla Shivakumar, Shilpa Sadanand, Bhavani Shankara Bagepally, Srinivas Brahmadevarahalli Krishnappa, Meera Purushottam, Palanimuthu Thangaraju Sivakumar, Sanjeev Jain, Mathew Varghese, Srikala Bharath

**Affiliations:** Department of Psychiatry, National Institute of Mental Health and Neurosciences, Bangalore 560029, India

## Abstract

*Objective*. To evaluate the association of Apolipoprotein E4 (ApoE4) in Alzheimer's dementia (AD) with comorbid diabetes mellitus (DM). *Methods*. The study included subjects with Alzheimer's dementia (AD) (*n* = 209), individuals with non-Alzheimer's dementia (nAD) (*n* = 122), individuals with parental history of AD (f/hAD) (*n* = 70), and control individuals who had normal cognitive functions and no parental history of dementia (NC) (*n* = 193). Dementia was diagnosed using International Classification of Diseases-10 revision (ICD-10) criteria. DM was assessed on the basis of self-report and/or use of antidiabetic medications. ApoE genotyping was done using sequence-specific primer polymerase chain reaction. *Results*. ApoE4 allele frequencies were highest among AD with comorbid DM (0.35) followed by AD without DM (0.25), nAD with DM (0.13), nAD without comorbid DM (0.12), and NC (0.08). Frequency of ApoE4 in persons with f/hAD was 0.13. The association of AD with co-morbid DM in ApoE4 carriers was more in comparison to NC with DM (OR = 5.68, *P* = 0.04). *Conclusion*. There is a significant association between AD with co-morbid DM and ApoE4 genotype.

## 1. Introduction

 With the rapid change in the demographic profile of India, the prevalence of chronic diseases of the elderly like dementia and DM type 2 is increasing. In India, over 3.7 million people aged 60 years and above suffer from dementia, predominantly of Alzheimer's type. Assuming that the current prevalence rate remains stable, these numbers are projected to increase to 6.35 million by 2025 [1]. Studies from India indicate that ApoE4 allele is significantly associated with AD, and it is a risk factor [2–5].

 There has been a pandemic rise of DM especially among Asian Indians. Currently 40.9 million Indians suffer from DM, and this is projected to increase to 69.9 million by 2025 [6]. Genetic predisposition and environmental factors interact to increase the risk of DM among Asian Indians. DM type 2 which accounts for 90% of DM is predominantly a polygenic disease with various loci identified at *PGC-1*α*, PC-1 (K121Q), IRS-2, MODY, and so forth* [7]. Many population-based cross-sectional studies [8] and prospective studies [9–13] have indicated that DM or abnormal glucose tolerance doubled the risk of AD (RR 1.37–2.27); though others have not confirmed this link [14]. Tripathi et al. [15], in a study from Northern India, reported DM as a risk factor for dementia; 33.3% of their sample with dementia had diabetes compared to 9% of the controls (*P* < 0.001). Other reports from Asia confirm the association between DM and AD in ApoE4 carriers. Peila et al. [16] in a large population-based study of Japanese American men found a significant association between DM, AD, and ApoE4 carriers; subjects with ApoE4 allele and DM had a relative risk of 5.5 for AD. Matsuzaki et al. [17] in a retrospective analysis of autopsies found a very significant positive association between hyperglycaemia, AD neuropathology, and ApoE4 status (OR 38.9).

We conducted a hospital-based cross-sectional study of dementia, to evaluate the interaction between AD and diabetes, as a function of the ApoE4 allele status.

## 2. Methods

### 2.1. Sample

Consecutive persons with dementia attending the Geriatric Clinic of the Department of Psychiatry in National Institute of Mental Health and Neurosciences (NIMHANS), Bangalore, and their consenting offspring were invited to participate in this study. This sample is part of a larger study of genetic and clinical correlates in dementia, and details about evaluation have been reported earlier [2]. In this paper, the study sample (*n* = 594) included AD (*n* = 209), nAD (*n* = 122), f/hAD (*n* = 70), and NC (*n* = 193). The nAD group consisted of vascular and mixed dementia (*n* = 46), frontotemporal dementia (*n* = 30), Lewy body dementia (*n* = 10), and other dementias (*n* = 36). Control subjects included genetically unrelated and cognitively intact age-matched subjects. The study was approved by the Ethics Committee of NIMHANS, and informed consent was collected prior to participation. All the subjects were evaluated using a standard protocol including general clinical evaluation, functional assessment (Everyday Abilities Scale for India, EASI [18]), neuropsychology testing (Hindi Mental Status Examination, HMSE [19]), laboratory investigations (haemogram, blood glucose levels), and genotyping and imaging (MRI scans using a 1.5 or 3 Tesla machine; or CT scan) whenever possible. Clinical evaluation was done by trained psychiatric residents and the diagnosis of dementia established using ICD-10 criteria. Further sub-typing was done by a psychiatric/neurology consultant. Their co-morbid DM was recorded based on laboratory results or self-reports, being on antidiabetic medication. Other participants were assessed using the same methodology and protocol.

Ten millilitres of venous blood was collected, and DNA was extracted using salting out method [20]. Polymerase chain reaction (PCR) was performed using sequence-specific primer PCR methodology [21]. The presence of 173-bp band was indicative of the specific ApoE haplotype. The results were corroborated with real-time PCR results for 10% of the samples in every experiment.

### 2.2. Statistical Methods

We compared ApoE4 frequency and DM status in AD, nAD, f/hAD, and NC. Statistical analysis was done using R software [22]. Comparisons between variables were done using chi-square test for categorical variables and unpaired *t*-test for continuous variables. Fisher's exact test was used when any of the values in the contingency table was less than 10. Odds ratios and *P* values were computed, and statistical significance was inferred if *P* < 0.05.

## 3. Results

Based on the sample selection, AD, nAD, and the NC were older than 60 years (61.14 ± 11.40 years; 61.73 ± 12.41 years; 60.35 ± 15.65 years, resp.). The AD, nAD, and NC group had crossed the age at risk for onset of DM. Results show that on average, 11.6% of our study group had developed DM. However, the prevalence of DM differed between the various groups, being—17.7% of the AD, 13.9% of the nAD, 6.2% of the NC, and 4.3% of the f/hAD (Table 1).

**Figure 1:**
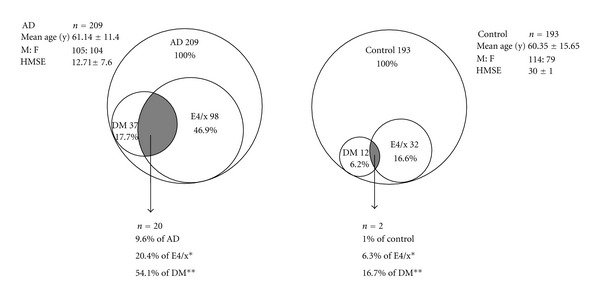
Venn diagram representation of AD and controls with their component distribution. Odds ratio comparing DM occurrence in ApoE4 carriers between AD and controls is 3.85 (*P* = 0.1*). Odds ratio comparing ApoE4-positive DM subjects between AD and controls is 5.68 (*P* = 0.04**).

ApoE4 carrier rates in AD (*n* = 98/209) were higher than age-matched NC (*n* = 32/193) (*P* < 0.0001, Table 1). The difference in ApoE4 carrier status between AD with comorbid DM and NC was significant (*P* = 0.0001). ApoE4 allele frequencies were highest among AD with co-morbid DM (0.35) followed by AD without DM (0.25), nAD with DM (0.13), nAD without co-morbid DM (0.12), and NC (0.08). The association of AD with co-morbid DM in ApoE4 carriers was more in comparison to NC with DM (OR = 5.68, *P* = 0.04, Figure 1).

There were 17 individuals in this study who were ApoE4 homozygotes—14 AD, 2 nAD, and one f/hAD. Among the 14AD with homozygous ApoE4, 6 (42.8%) individuals were receiving treatment for DM. In comparison, there were 84 AD who were ApoE4 heterozygotes, of whom only 14 had DM (16.6%). Homozygous ApoE4 carriers had significantly greater comorbidity of DM and AD (*P* = 0.035).

## 4. Discussion

The present cross-sectional study evaluates the relationship between ApoE4 status, AD and DM in a sample from southern India. The study also confirms the significant association between ApoE4 allele and AD in the Indian population [2–5]. The ApoE4 frequency however was not associated with the age of onset of AD, or the degree of cognitive and functional ability as assessed by HMSE and EASI.

The study also reiterates the increased association of AD and DM (*P* = 0.0004) established by other studies [8–13, 15]. Interestingly, AD individuals with DM were more likely to be ApoE4 carriers compared to NC with DM (OR 5.68). The ApoE4 allele frequency was also significantly high in this group (0.35). The current study indicates a need to evaluate the role of ApoE4 as a risk factor for DM in future. Earlier research of ApoE4 as a risk factor for DM has yielded inconsistent results. Study in Mexican Americans [23] did not find any difference in ApoE4 frequency between diabetics and nondiabetics. It is interesting to note that Xu et al. [10] found a significant association between borderline DM and AD only in non-ApoE4 carriers. Assessment of the ApoE4 status in DM without AD in the Indian population would be able to provide some clarity towards this. Individuals with ApoE4 allele if found to have an increased risk to DM in their middle age, the combined presence of ApoE4 and DM might further increase the risk of AD later. Testing of this hypothesis was not feasible in the present study as it was a cross-sectional one from a Geriatric Clinic which caters to the needs of elderly with dementia. A prospective controlled followup study of the individuals with DM with and without ApoE4 allele into old age is needed to establish the implications of this association of ApoE4, AD and DM.

Lack of prospective design, detailed assessment of DM and other validating phenotypes of AD like neuropsychological assessment, and imaging techniques for all the subjects are the limitations of the study. Reasonable sample size, methodological rigor in diagnostic assessment, and an attempt to correlate two conditions of increasing concern in India, that is, diabetes and AD in the context of ApoE4 allele are the strengths of the work.

Translational aspect of this association if proven is very important. In the Indian context, prevalence of diabetes is on the rise. Lifestyle and diet control measures to prevent DM in ApoE4-positive individuals especially with homozygosity in middle age may modify/reduce the risk of Alzheimer's dementia.

## Figures and Tables

**Table 1:** Clinical profile and ApoE polymorphism in the study population.

	Alzheimer's dementia (AD) (*n* = 209)	Non-Alzheimer's dementia (nAD) (*n* = 122)	Individuals with parental history of AD (f/hAD) (*n* = 70)	Control individuals with no parental history of dementia (NC) (*n* = 193)	*P* value (comparing AD and NC)	*P* value (comparing nAD and NC)
Mean age in years	61.14 ± 11.40	61.73 ± 12.41	37.27 ± 8.87	60.35 ± 15.65	0.56^++^	0.41^++^
M : F	105 : 104	75 : 47	51 : 19	114 : 79	0.076*	0.67*
DM, *n* (%)	37 (17.7)	17 (13.9)	3 (4.3)	12 (6.2)	0.0004*	0.02*
HMSE	12.71 ± 7.60	14.19 ± 8.19	30 ± 1	30 ± 1	**<0.0001** ^++^	**<0.0001** ^++^
EASI	8.41 ± 2.76	8.19 ± 3.03	0	0	**<0.0001** ^++^	**<0.0001** ^++^
E2E2, *n* (%)	0 (0)	1 (0.8)	0 (0)	0 (0)	1^+^	0.39^+^
E2E3, *n* (%)	11 (5.3)	11 (9)	5 (7.1)	23 (11.9)	0.017*	0.42*
E3E3, *n* (%)	100 (47.8)	82 (67.2)	48 (68.6)	138 (71.5)	<0.0001*	0.42*
E2E4, *n* (%)	13 (6.2)	4 (3.3)	0 (0)	2 (1)	**0.007** ^+^	0.21^+^
E3E4, *n* (%)	71 (34)	22 (18)	16 (22.9)	30 (15.5)	<0.0001*	0.56*
E4E4, *n* (%)	14 (6.7)	2 (1.6)	1 (1.4)	0 (0)	**0.0001** ^+^	0.15^+^
Total ApoE4 carrier, *n* (%)	98 (46.9)	28 (23)	17 (24.3)	32 (16.6)	<0.0001*	0.16*
ApoE4 carrier with DM, *n* (%)	20 (9.6)	4 (3.3)	0 (0)	2 (1)	**0.0001** ^+^	0.21^+^

HMSE: Hindi Mental Status Examination (indicates level of cognitive function; highest score possible = 31).

EASI: Everyday Abilities Scale for India (indicates level of functional disability, highest score possible = 12).

*P* values calculated by unpaired *t*-test^++^, chi-square test*, and Fisher's exact test^+^.
